# Potential of Mesenchymal Stem Cell-Derived Exosomes as a Novel Treatment for Female Infertility Caused by Bacterial Infections

**DOI:** 10.3389/fmicb.2021.785649

**Published:** 2022-01-27

**Authors:** Marzieh Zohrabi, Laleh Dehghan Marvast, Mahin Izadi, Seyed Alireza Mousavi, Behrouz Aflatoonian

**Affiliations:** ^1^Research and Clinical Center for Infertility, Yazd Reproductive Sciences Institute, Shahid Sadoughi University of Medical Sciences, Yazd, Iran; ^2^Department of Reproductive Biology, School of Medicine, Shahid Sadoughi University of Medical Sciences, Yazd, Iran; ^3^Andrology Research Center, Yazd Reproductive Sciences Institute, Shahid Sadoughi University of Medical Sciences, Yazd, Iran; ^4^Infectious Diseases Research Center, Shahid Sadoughi Hospital, Shahid Sadoughi University of Medical Sciences, Yazd, Iran; ^5^Stem Cell Biology Research Center, Yazd Reproductive Sciences Institute, Shahid Sadoughi University of Medical Sciences, Yazd, Iran; ^6^Department of Advanced Medical Sciences and Technologies, School of Paramedicine, Shahid Sadoughi University of Medical Sciences, Yazd, Iran

**Keywords:** antimicrobial effects, mesenchymal stem cells, MSC-derived exosomes, antibacterial properties, *Neisseria gonorrhoeae*, *Chlamydia trachomatis*, female infertility

## Abstract

*Neisseria gonorrhoeae* and *Chlamydia trachomatis* are the most common causes of bacterial sexually transmitted diseases (STDs) with complications in women, including pelvic inflammatory disease (PID), ectopic pregnancy, and infertility. The main concern with these infections is that 70% of infected women are asymptomatic and these infections ascend to the upper female reproductive tract (FRT). Primary infection in epithelial cells creates a cascade of events that leads to secretion of pro-inflammatory cytokines that stimulate innate immunity. Production of various cytokines is damaging to mucosal barriers, and tissue destruction leads to ciliated epithelial destruction that is associated with tubal scarring and ultimately provides the conditions for infertility. Mesenchymal stem cells (MSCs) are known as tissue specific stem cells with limited self-renewal capacity and the ability to repair damaged tissues in a variety of pathological conditions due to their multipotential differentiation capacity. Moreover, MSCs secrete exosomes that contain bioactive factors such as proteins, lipids, chemokines, enzymes, cytokines, and immunomodulatory factors which have therapeutic properties to enhance recovery activity and modulate immune responses. Experimental studies have shown that local and systemic treatment of MSC-derived exosomes (MSC-Exos) suppresses the destructive immune response due to the delivery of immunomodulatory proteins. Interestingly, some recent data have indicated that MSC-Exos display strong antimicrobial effects, by the secretion of antimicrobial peptides and proteins (AMPs), and increase bacterial clearance by enhancing the phagocytic activity of host immune cells. Considering MSC-Exos can secrete different bioactive factors that can modulate the immune system and prevent infection, exosome therapy is considered as a new therapeutic method in the treatment of inflammatory and microbial diseases. Here we intend to review the possible application of MSC-Exos in female reproductive system bacterial diseases.

## Introduction

Today, with the huge concern regarding antibiotic resistance and due to absence of an effective vaccine, researchers are looking for suitable alternatives to solve this problem (Hosseiniyan Khatibi et al., 2020; Russell et al., 2020). Mesenchymal stem cells (MSCs) are defined as undifferentiated renewable cells. These cells can be isolated from different tissues including bone marrow, cord blood, skin, fallopian tube, liver, lungs, endometrium, testis, amnion, ovary, and adipose tissue (Akyash et al., 2016a,b; Sadeghian-Nodoushan et al., 2016; Zhang et al., 2016; Akyash et al., 2020; Hoseini et al., 2020). Moreover, there are reports indicating the generation of MSCs from pluripotent human embryonic stem cells (hESCs) (Akyash et al., 2016a; Javidpou et al., 2021). The therapeutic potentials of MSCs are accomplished through three mechanisms. The first is differentiation into multiple cell types, which provides the condition for repairing and replacing damaged tissues. The second is that MSCs migrate to injured tissues due to chemical gradients. The third mechanism is the most important mechanism due to secretion of bioactive factors (Vizoso et al., 2017). Moreover, MSCs are able to secrete nanoparticles called exosomes that from by fusion of the cell membrane of multivesicular and are considered as extracellular vesicles (EVs). EVs according to *International Society for Extracellular Vesicles* (ISEV) are divided into three classes based on their size and origin, which include exosomes, microvesicles (MVs), and apoptotic bodies; (a) exosomes with various in size of 30–150 nm originate from multivesicular bodies (MVBs), (b) microvesicles in size of 150–1000 nm, (c) apoptotic bodies with a wide size distribution of 50–2000 nm (Gurunathan et al., 2019; Akbari and Rezaie, 2020; Rezaie et al., 2021). Studies show that MSCs exert their paracrine effects by secreting exosomes which are known by other names including nanoparticles, exosome-like vesicles, dexosomes, prostasomes, and tolerosomes (Zhang et al., 2020; Rezaie et al., 2021). Exosomes are transitional vesicles that release into the extracellular space through fusion with the cell membrane, which can reach distance target cells and affect their function and activity (Kowal et al., 2014). MSC-derived exosomes (MSC-Exos) are able to secrete cytokines, chemokines, and growth factors, proteins, mRNA, non-coding RNA, and bioactive lipids that could elicit a wide range of physiological activities (Harrell et al., 2019; Adib et al., 2020b,a; Yuan et al., 2020). Moreover MSC-Exos are considered as an innovative therapeutic tool to treat bacterial infections, consistent with their unique properties (Park et al., 2019). *Neisseria gonorrhoeae* (*N. gonorrhoeae*) and *Chlamydia trachomatis* (*C. trachomatis*) are gram-negative bacteria that are both considered as obligate human pathogens (Chen et al., 2018). Due to the pathogenesis of *N. gonorrhoeae* and *C. trachomatis* and the ability of these bacteria to cause chronic infections and, on the other hand, considering the side effects of antibiotic resistance and the absence of effective vaccines, new treatment strategies are needed to repair damaged epithelial cells of fallopian tube (FT) in these infections. As regards conditioned medium (CM) or MSC-Exos contain growth factors, antimicrobial peptides/proteins (AMPs) and cytokines have immunosuppression properties on innate and adaptive immune responses *via* direct and indirect mechanisms. In addition, CM and MSC-Exos have other therapeutic potentials including anti-apoptotic activity, wound healing, tissue repair, antiscarring, and angiogenesis regulation (Burlacu et al., 2013; Williams et al., 2013; Vizoso et al., 2017; Adib et al., 2020b,a), Here, we intend to review the application of MSC-Exos in female reproductive system bacterial diseases.

## Antimicrobial Effects of Mesenchymal Stem Cells

Many studies have shown that MSCs display antimicrobial features by secretion of AMPs and regulation of immune responses (Krasnodembskaya et al., 2010; Koniusz et al., 2016). These antimicrobial effects of MSCs are mediated *via* direct and indirect mechanisms (Russell et al., 2020). MSCs directly interact with pathogens by secreting AMPs, including lipocalin 2, cathelicidin, β-defensin 2, and hepcidin, thereby playing an important role in increasing bacterial clearance (Marrazzo et al., 2019; Chow et al., 2020). While MSCs are exposed to pathogenic factors, including pathogen-associated molecular patterns (PAMPs), lipopolysaccharide (LPS), and damage-associated molecular patterns (DAMPs) *via* toll-like receptors (TLRs), caused a change in their proliferation, differentiation, migration, and secretory factors (Marrazzo et al., 2019; Hosseiniyan Khatibi et al., 2020). The immunomodulatory effects of MSC-Exos are mainly due to inhibition of T cells proliferation and conversion of these cells to regulatory T cells (Tregs) and also through reprogramming of M1 macrophage cells to M2 phenotype that these immunomodulatory and anti-inflammatory effects of MSC-Exos lead to tissue repair and healing (Xie et al., 2020; Arabpour et al., 2021).

### Direct Mechanisms

**FIGURE 1:**
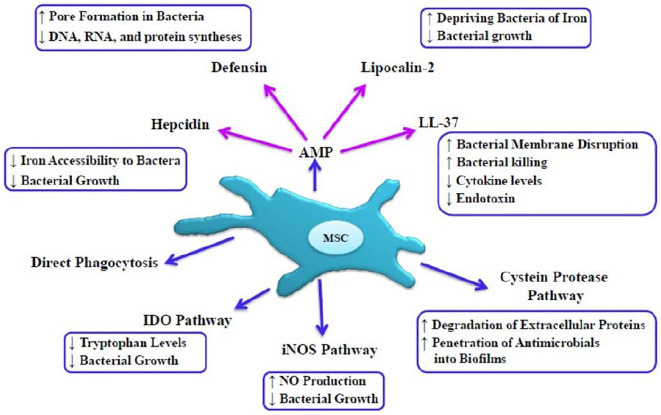
Direct mechanisms of MSC-mediated bacterial killing, MSC exert direct antibacterial effects due to host defense peptides or AMPs. MScs can mediate bacterial killing by disrupting the integrity of the bacterial membrane, creating pores in the bacterial membrane, preventing of iron uptake, inhibiting biofilm formation, depleting tryptophan in microorganisms, and halting the growth of bacteria. IDO pathways, Indoleamine 2,3 dioxygenase; iNOS pathways, inducible nitric oxide synthase; NO, nitric oxide.

Antimicrobial peptides and proteins secreted from the MSC directly play important roles in the bacteria clearance from different pathways, including inhibition in the synthesis of DNA and RNA, disruption of membrane integrity, and inhibition of bacterial growth through disruption in iron uptake (Brogden, 2005; Hosseiniyan Khatibi et al., 2020). AMPs are produced as the first line of defense of innate immunity against a wide range of microorganisms, including bacteria, viruses, and fungi (Diamond et al., 2009). Families of MSCs-derived AMPs listed in Figure 1 are mainly studied including cathelicidin, β-defensin-2, lipocalin 2, and Hepcidin (Alcayaga-Miranda et al., 2017; Russell et al., 2020).

#### Cathelicidin

One of the important antibacterial peptides is the cathelicidin family, which recruits monocytes, neutrophils, and macrophages (Krasnodembskaya et al., 2010). LL-37 is a factor of this family that is an essential part of the innate immune system that exerts its antibacterial effect by disrupting the integrity of the bacterial membrane and neutralizing LPS (Krasnodembskaya et al., 2010; Thennarasu et al., 2010). This factor has also been shown to play an important role in regulating inflammatory responses, inducing tissue repair and healing as well as anti-apoptotic and angiogenic effects (Oliveira-Bravo et al., 2016; Yang et al., 2020), and in a mouse model of septicemia provided protection against endotoxin shock (Yagi et al., 2020). Johnson et al. (2017), in a study of the antibacterial effects of MSC administration in chronic infections associated with biofilms in mouse and dog models, stated that i.v. administration of activated MSCs induce the killing of bacteria by secretion of cathelicidin, and this effect was increased by antibiotics.

#### β-Defensin 2

β-defensin 2 play important roles in innate and adaptive immunity against microbial and exert its antibacterial effect by creating pores in the bacterial membrane and destroying the integrity of the membrane and leaking intracellular contents, as well as inhibiting protein, DNA, and RNA syntheses (Laverty et al., 2011; Méndez-Samperio, 2013). A study showed that MSCs secrete the antimicrobial peptide of β-defensin 2 through the TLR-4 signaling pathway after exposure to *Escherichia coli* (Sung et al., 2016). The bacteriostatic potential of this peptide is mainly against gram-negative bacteria and with a lower antibacterial potential against gram-positive bacteria (Harder and Schröder, 2005).

#### Lipocalin 2

Lipocalin 2 is secreted by various cells including neutrophils, macrophages, epithelial cells, and MSCs in response to inflammatory conditions, which plays an important role in the antibacterial defense of the innate immunity (Dahl et al., 2018). After exposure of MSCs to pathogenic factors lead to the secretion of a large amount of lipocalin 2, this peptide binds to siderophore, as an iron chelator of bacteria, which in turn prevents iron uptake and subsequently reduces bacterial growth (Goetz et al., 2002; Flo et al., 2004). Harman et al. (2017) reported that MSCs derived from the peripheral blood of healthy horses by the secretion of AMPs, including Cystatin C, elafin, lipocalin, and cthelicidin, through disturbance in membrane integrity, the growth of bacteria (*E. coli* and *Staphylococcus aureus*) was inhibited .

#### Hepcidin

Hepcidin is secreted by hepatocytes, renal epithelial cells, as well as by macrophages and MSCs in inflammatory conditions, which plays an important role in the systemic regulation of iron homeostasis (Kulaksiz et al., 2004, 2005; Esfandiyari et al., 2019). Hepcidin is an antibacterial peptide of the innate immune system that is primarily induced by the IL-6, LPS, and TLR-4 which sequesters bacterial siderophores, and therefore restricts iron availability and as a result inhibits bacterial growth (Ganz and Nemeth, 2012; Michels et al., 2017). Two isoforms of hepcidin are known, including hepcidin 20 and 25, both of which have antibacterial properties (Maisetta et al., 2010).

#### Inducible Nitric Oxide Synthase Pathway

Mesenchymal stem cells and macrophages activated by LPS, pro-inflammatory cytokines, and interferons (IFN) cause the expression of inducible nitric oxide synthase (iNOS), which in turn iNOS produces nitric oxide (NO) from the amino acid L-arginine inside these cells. Production of NO in this way halts the growth of microorganisms inside macrophages and MSCs (Bogdan, 2015; Yang et al., 2016).

#### Cysteine Proteases

Studies show that MSC-Exos contain a variety of proteases, including cysteine proteases, which impact the stability of bacterial biofilms by degrading extracellular proteins, and thereby provide conditions for antimicrobials penetration into biofilms and also increase the effectiveness of antibiotics tolerated by biofilms previously (Matsumoto et al., 1999; Marx et al., 2020).

#### Indoleamine 2,3 Dioxygenase

Indoleamine 2,3 dioxygenase (IDO) is the most important enzyme in the kynurenine pathway (KP), which is primarily responsible for the degradation of the tryptophan amino acid, which MSCs mainly express this enzyme in response to the stimulatory effect of INFγ (Däubener et al., 2009; Croitoru-Lamoury et al., 2011). Depletion of tryptophan in microorganisms due to IDO impairs protein synthesis and disrupts cell division (Frumento et al., 2002; Meisel et al., 2011). IDO has also been shown to induce immunomodulatory effects by inhibiting T cells proliferation and modulating the function of B, T cells, and natural killer (NK) cell (Poormasjedi-Meibod et al., 2013).

### Indirect Mechanisms

**FIGURE 2:**
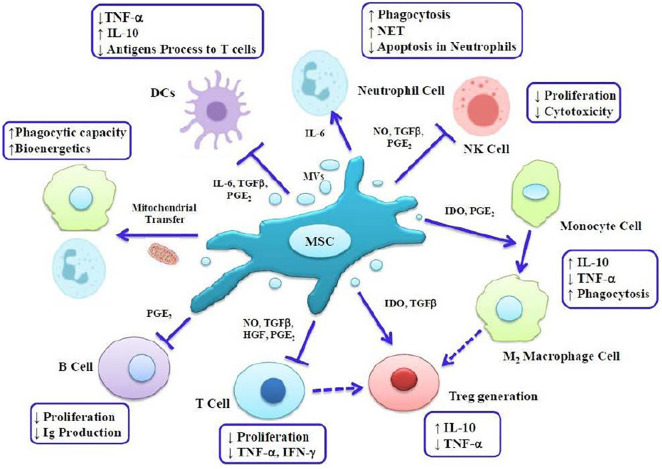
The antibacterial effects of MSC can be indirectly mediated by increasing phagocytic activity of macrophages and neutrophils. These cells can also induce immunomodulatory effects by modulating immune responses and regulating cytokine homeostasis. MVs, microvesicles; DC, dendritic cell; NKC, natural killer cell; NET, neutrophil extracellular trap; IFN, interferon; TNF, tumor necrosis factor; NO, nitric oxide; PGE2, prostaglandin E2; TGF, transforming growth factor; IDO, indoleamine 2,3 dioxygenase; Treg, T regulatory cell; Ig, immunoglobulin.

The antibacterial effects of MSCs can be indirectly mediated by increasing phagocytic activity of macrophages and neutrophils (Hosseiniyan Khatibi et al., 2020). These cells can also induce immunomodulatory effects mentioned in Figure 2 by modulating immune responses and regulating cytokine homeostasis and reducing immune cells transfer into the damaged organ, and thereby provide the conditions for tissue remodeling and healing. Moreover MSC-Exos perform their major immunomodulatory effects by inhibiting T cell proliferation and converting these cells to Tregs as well as reprogramming M1 macrophages to the M2 phenotype (Riazifar et al., 2019; Hoseini et al., 2020; Hosseiniyan Khatibi et al., 2020; Liu W. et al., 2020). These cells and their exosomes can also inhibit the proliferation and function of B cells, natural killer cells (NKC), and dendritic cells (DC). MSCs can induce both bacterial clearance and immunomodulatory effects, which are dependent on inflammatory signals in the environment (Fan et al., 2019; Xie et al., 2020). MSCs increase immune responses during the early phases of inflammation such that in addition to the migration of neutrophils to sites of inflammation, MSCs induce lymphocyte and M1 macrophages, through the production of chemokines. In fact, the stimulatory effects of mesenchymal cells on immune cells occur when these cells encounter insufficient levels of proinflammatory cytokines such as TNF and IFN-γ, while MSCs and MSC-Exos provide conditions for immunosuppressing during exposure to high levels of inflammatory cytokines through polarization to anti-inflammatory cells, M2 macrophages, and Tregs (Raicevic et al., 2010; Bernardo and Fibbe, 2013; Song et al., 2017; Xie et al., 2020). Thus, MSCs can activate both phenotypes of macrophages and provide a balance between inflammatory and anti-inflammatory responses through interaction with the immune system and thereby provide the condition for maintaining integrity and homeostasis of tissue (Liu W. et al., 2020; Xie et al., 2020). MSCs inhibit proliferation and function of T cells by secreting factors such as nitric oxide (NO), IDO, prostanglandin-E2 (PGE2), transforming growth factor (TGF)-β, and interleukin (IL)-10 (DelaRosa and Lombardo, 2010).

Kol et al. (2014) in the study of effects of adipose-derived MSCs on intestinal microbes (*Salmonella typhimurium* and *Lactobacillus acidophilus*) concluded that these cells could increase the expression of key immunomodulatory genes including COX2, IL-6, and IL-8, as well as increase the secretion of PGE2, IL-6, and IL-8, and they also found that exposure of MSCs to *S. typhimurium* increased the capacity of these cells to inhibit T cell proliferation *via* PGE2. MSC-Exos also exert their immunomodulatory effects through their RNA and proteins (Lo Sicco et al., 2017). Song et al. stated that exosomal miR-146a is an anti-inflammatory micro-RNA that is transferred into macrophages and leads to polarization to M2 phenotype and ultimately increases survival in sepsis models of mice (Song et al., 2017).

It has also been shown that MSCs inhibit T cell activity by inhibiting the function, differentiation, and maturation of dendritic cells (DCs) (Aggarwal and Pittenger, 2005). DCs are the main cells of the immune system which present antigens to T cells and are able to express high levels of co-stimulatory molecules and thereby effectively induce immune responses; thus MSCs and MSC-Exos can lead to inhibition of T cells function and development of Tregs by inducing an inhibitory effect on DCs (Aggarwal and Pittenger, 2005; Jiang et al., 2005). Moreover, MSCs lead to recruitment and stimulation of polymorphonuclear (PMN) cells such as neutrophils, by secreting IL-6 and IL-8 (Brandau et al., 2014). Neutrophils induce the killing of microorganisms by phagocytosis and internalization of them into the phagolysosome, and it has also been shown that these cells, through mechanism of neutrophil extracellular traps (NETs), immobilize microorganisms to prevent their spread in the environment (Hirschfeld, 2014; Jackson et al., 2016). Studies show that MSCs and MSC-Exos not only increase phagocytosis activity of neutrophils but also protect neutrophils from apoptosis (Harrell et al., 2019; Qian et al., 2021). In addition, research showed that direct co-culture of MSCs and their exosomes with macrophages induce mitochondrial transfer from MSCs to macrophages *via* formation of structures called tunneling nanotubes (TNT) that leads to increase in the phagocytic activity of macrophages and improvement in their bioenergetics (Hirschfeld, 2014; Qian et al., 2021).

## Pathogenesis of *N*. *Gonorrhoeae* and *C*. *Trachomatis* in the Female Reproductive Tract

*Neisseria gonorrhoeae* and *Chlamydia trachomatis* are gram-negative bacteria that are both considered obligate human pathogens, and they are known as the most common cause of sexually transmitted diseases (STDs) (Dehghan Marvast et al., 2016, 2018; Chen et al., 2018; Lenz and Dillard, 2018). *N. gonorrhoeae* mainly affects the mucous membranes of female reproductive tracts. This infection starts from the lower reproductive tract including the vagina and ectocervix and can spread to the upper female genital tract (endometrium and fallopian tubes) (Lenz and Dillard, 2018). *Chlamydia* is also an intracellular pathogen that infects the epithelial cells of the endocervix in women and the urethra in men (O’Connell and Ferone, 2016). During its evolutionary cycles, *Chlamydia* forms structures called elementary bodies (EBs) and reticulate bodies (RBs). EBs are infective forms that are metabolically inactive, but after chlamydia enters the host cell, EBs convert to RBs that are metabolically active but non-infectious and are considered as the replicating form of the bacteria (Brunham and Rey-Ladino, 2005). *N. gonorrhoeae* and C. trachomatis infections can be symptomatic or asymptomatic and without treatment lead to complications such as pelvic inflammatory disease (PID), obstruction of FT, tubal scarring, and loss of ciliated cells function in these areas (Dehghan Marvast et al., 2017; Tsevat et al., 2017). Studies have reported that *N. gonorrhoeae* attach to non-ciliated cells through pili and Opa proteins in FT but lead to loss of ciliated cells function and eventually the death of these cells (Edwards and Apicella, 2004; Quillin and Seifert, 2018). Various studies have linked the death of these cells to the presence of toxic factors in bacteria, including lipopolysaccharide (LPS) and lipooligosaccharide (LOS), which induce the host immune system (Gregg et al., 1981; Christodoulides, 2019; Gulati et al., 2019). In the gonococcal infections following exposure to pathogen associated molecular patterns (PAMPs), the secretion of cytokine TNF is one of the first responses of the host immune system (Patrone and Stein, 2007). One study reported that increase in the concentration of TNF was associated with decrease in function of ciliated cells (McGee et al., 1999). On the other hand, studies have reported that the reduction in ciliated cell activity and death of these cells during gonococcal infections has been attributed to the induction of apoptosis in FT epithelial cell by the TNF cytokine (Edwards and Apicella, 2004; Morales et al., 2006). Evidences also are showed that other factors, including IL-1, IL-6, IL-8, monocyte chemoattractant protein-1 (MCP-1), and granulocyte macrophage colony-stimulating factor (GM-CSF) secreted during gonococal infections (Maisey et al., 2003; Velasquez et al., 2012). Moreover, one study reported that the levels of cytokines IL-2 and IL-12 were rapidly upregulated during exposure to *N. gonorrhoeae* infection, such that IL-2 was associated with lymphocyte proliferation, while IL-12 increased IFNγ production by lymphocytes and NKC (Rarick et al., 2006). Although magnitude Th17 responses in gonococcal infections lead to the release of IL-17 and the recruitment of neutrophils, the relative resistance of *N. gonorrhoeae* to neutrophil function and the lack of an effective response to pathogen clearance have been reported (Witt et al., 1976; Liu et al., 2012). Moreover, immune responses in *C. trachomatis* infection include activation of Th1 and proinflammatory cytokines IL-2, IL-6, TNF, and INF-γ (Arno et al., 1990). One study reported that INF-γ levels in endocervical secretions of women with C. trachomatis infection were five times higher than uninfected women (Sellami et al., 2014). Also, studies show that TLR-2 and TLR-4, which are increased in *C. trachomatis* infection, play an important role in inducing innate and acquired immune responses (Agrawal et al., 2011; Lovett and Duncan, 2019).

But although lymphocyte proliferative responses in gonococcal infections are increased compared to healthy individuals, these immune responses cannot provide strong protection against recurrence of the infection (Zhu et al., 2012). Moreover, *N. gonorrhoeae* are able to manipulate and effect the function of host immune cells, so that gonococcal infections have been shown to exert immunosuppressive signaling by inhibiting the proliferation of DCs, T cells, and B cell (Manicassamy et al., 2009; Escobar et al., 2018). On the other hand, *N. gonorrhoeae* induces the expression of immunosuppressive cytokines such as TGF-β and IL-10 so that it has been stated that *N. gonorrhoea* suppresses the activity of Th1 and Th2 by inducing the expression of TGF-β (Mascellino et al., 2011).

Evidence suggests that the immune responses generated during the pathogenesis of Neisseria and Chlamydia are polarized toward cytotoxic responses and provide the conditions for obstruction and scaring in FT (Menon et al., 2015; Jefferson et al., 2021). According to research, different mechanisms are involved in inducing infertility following *N. gonorrhoeae* and *C. trachomatis* infection. The first mechanism involves the ascension of the infection to the upper reproductive tract (Hafner, 2015). The second mechanism involves the persistence of the infection, which leads to long-term pathological immune responses and thus provides the conditions for damage to the epithelial cells of FT (Batteiger et al., 2010). It has also been suggested that treatment failures by antibiotics lead to recurrence of the infection and the development of infertility *via* persistence of infection (Menon et al., 2015). The third mechanism involves the secretion of cytokines from pathogen-infected epithelial cells, which induce proinflammatory immune responses that lead to severe epithelial cell damage and fibrosis or scarring following repair mechanisms by infiltrating fibroblasts (Darville, 2021). Since that salpingitis induced by *N. gonorrhoeae* and *C. trachomatis* infections leads to pathological immune responses and induces infertility, it is necessary to create a good balance between immune activation and immune suppression.

## Therapeutic Potential of Mesenchymal Stem Cell-Derived Exosomes on Salpingitis Induced by *N. Gonorrhoeae* and *C. Trachomatis* Infections

Due to the unique life cycles of *N. gonorrhoeae* and *C. trachomatis*, the major PID caused by these infections are chronic, so antibiotic therapy is less effective, which often leads to persistence of the infection and reinfection (Chen et al., 2020). Various studies listed in Table 1 have reported the positive effects of MSC-Exos in the treatment of gynecological diseases (Sun et al., 2019; Zhang et al., 2019; Liu C. et al., 2020; Xin et al., 2020; Zhao et al., 2020; Lee et al., 2021; Liao et al., 2021). Today, MSC-Exos are used in cell therapy, regenerative medicine, auto-immune, and microbial disease due to their unique properties such as high proliferative capacity, easy isolation, and secretion of bioactive factors, as well as having anti-apoptotic, antimicrobial, antiscarring, tissue repair, and wound healing effects (Ha et al., 2020; Raghav et al., 2021).

**TABLE 1:** Antibacterial and Immunomodulatory Effects of MSCs and MSC-Exos in *in vitro* and *in vivo* studies.

Study type	Source of MSC	Outcomes	References
***In vivo*:** Mouse and dog models of chronic infections	AT-MSC	↑ Cathelicidin secretion ↑ Clearance of bacteria ↑ Monocyte recruitment ↑ M2 phenotype ↑ Neutrophil bacterial Phagocytosis	Johnson et al., 2017
***In vivo*:** Murine Cystic fibrosis	BM-MSC, AT-MSC	↑ Enhance antibiotic sensitivity ↑ Capacity to kill bacteria (*Pseudomonas aeruginosa*, *Staphylococcus* *aureus*) ↑ LL-37	Sutton et al., 2017
***In vitro*:** Bacterial growth in Equine model	BM-MSC, AT-MSC, EM-MSC	↓ Growth of *E. coli* ↑ Lipocalin-2 expression ↑ MCP-1, IL-6, IL-8, and CCL5	Cortés-Araya et al., 2018
***In vitro* and *In vivo*:** Murine sepsis model	BM-MSC	↓ Genes expression of apoptosis ↓ Genes expression of Pro-inflammatory cytokine ↑ Antibacterial peptides ↑ Anti-inflammatory cytokines ↑ Animal survival rates ↑ Bacterial clearance (Staphylococcal enterotoxin B)	Saeedi et al., 2019
***In vitro*:** Chronic skin wounds in Equine model	PB-MSC	↓ Growth of *E. coli* and *S. aureus* biofilms ↑ Cystatin C, elafin, lipocalin, cthelicidin	Harman et al., 2017
***Ex vivo*:** Acute Lung Injury in Mice	HU-MSC	↑ Keratinocyte growth factor (KGF) ↓ Influx of neutrophils ↓ Lung protein permeability ↓ Pulmonary edema	Zhu et al., 2014
***In vivo*:** Chronic inflammation (*Staphylococcus aureus*) of the ovaries in mice	BM-MSC	↓ Leukocyte infiltration in ovaries ↓ Number of atretic follicles ↑ Ovary morphological parameters ↓ Apoptotic oocytes ↑ Pregnancy rate	Volkova et al., 2017
***In vivo*:** Chronic salpingitis (*E. coli*) model in rabbits	WJ-MSC	↓ TNF-α ↑ Oviductal glycoprotein ↑ Repaired the structure of the tubal epithelium ↑ Pregnancy rates	Li et al., 2017
***In vivo*:** Chronic salpingitis (*Chlamydia trachomatis*) murine model	hUC-MSC	↓ Macrophage infiltration ↑ IL-10 ↓ FT cell apoptosis (Caspase-3) ↑ Pregnancy rate	Liao et al., 2019
***In vitro*:** Human Fetal Liver	FL-MSC-Exos	↓ Proliferation, activation, and cytotoxicity of NK cells *via* TGFb	Fan et al., 2019
***In vivo*:** Intrauterine adhesions in a female rat model	UC-MSCs-EVs	↓TNF-α, ↓TGF-β ↓IL-1, ↓IL-6 ↓RUNX2, ↓Fibrosis ↓collagen-I ↓VEGF ↓IUA	Ebrahim et al., 2018
***In vivo*:** Premature ovarian insufficiency model mice	hU-MSC-Exos	↑Restored ovarian phenotype and function ↑ovarian cells proliferation ↑exosomal miR-17-5P ↓SIRT7 expression	Ding et al., 2020
***In vitro***: inflammation in endometrial cells of equine models	A-MSC- MVs	↓Apoptosis rate ↓Pro-inflammatory gene expression ↓Pro-inflammatory cytokines secretion	Perrini et al., 2016
***Ex vivo*:** Lung injury models in mice	BM-MSC-EV	↑M2 macrophage marker expression ↑Phagocytic macrophage Phenotype ↑Mitochondrial transfer to macrophage ↓Inflammation and lung injury	Morrison et al., 2017
***In vitro*:** Asthma in human	BM-MSC-Exos	↑IL-10 ↑TGF-β1 ↑Immunosuppression capacity of Tregs	Du et al., 2017

*MSC, mesenchymal stem cell; AT-MSC, adipose tissue-MSC; HU-MSC, human-MSC; BM-MSC, bone marrow-MSC; EM-MSC, endometrium-MSC; PB-MSC, peripheral blood-MSC; FL-MSC, fetal liver-MSC; WJ-MSC, wharton’s jelly; hUC-MSC, human umbilical cord-MSC; A-MSC, amniotic-MSC; MCP-1, monocyte chemoattractant protein-1; CCL5, chemokine ligand-5; FT, Fallopian tube; IUAs, intrauterine adhesions.*

### Exosomes Isolation of Mesenchymal Stem Cell

Various techniques are used to separate exosomes from MSCs, including ultracentrifugation, ultrafiltration, precipitation, immunological separation, chromatography, and nanoFACS (Théry et al., 2018; Rezaie et al., 2021). However, each of these methods has advantages and disadvantages, and studies have reported that the ultracentrifugation method is the most common standard method for isolating exosomes (Momen-Heravi et al., 2013). But Klymiuk et al. (2019) in their study stated that the ultrafiltration method had higher results and efficiencies in size-based isolation compared to the ultracentrifugation method, and a 50-fold increase in concentration and less time for isolation compared to the ultracentrifugation method was reported. On the other hand, due to several overlapping features between exosomes and viruses such as size, shape, density, and biogenesis, Rezaie et al. (2021) reported that nanoFACS and immunological methods are more suitable for isolating exosomes from viruses in infected samples. Moreover, in most studies it has been stated that in order to achieve better specificity and recovery in the separation of EV or EV subtypes, the use of a combination of techniques or additional techniques is recommended (Nikfarjam et al., 2020; Liangsupree et al., 2021).

### Advantages and Limitations of Mesenchymal Stem Cells-Derived Exosomes Application

Various studies have shown the superiority of using exosomes rather than MSCs. The risk of tumor formation has not been reported in exosome-based therapies, while the tumorigenic risk in MSC-based therapies has been observed in several studies (Mendt et al., 2019; Wei et al., 2021). In addition to the fact that lower side effects of exosomal therapy than mesenchymal transplantation have been reported in various studies, Sun et al. (2015) have reported increased expression of HLA and immunological rejection in MSCs transplantation. In addition, studies have shown that exosomes are not affected by apoptotic processes and cell death due to their non-cellular nature, and therefore their stability is greater in the damaged area (Lou et al., 2017; Wang et al., 2021). Exosomes are also less expensive to produce than MSCs and are more stable to store and easier for storage and, recently, it has received more attention than cell-based therapy due to the ability of exosomes to transport therapeutic biomolecules and facilitate repair of the damaged site (Babaei and Rezaie, 2021). Despite the advantages of exosome therapy and its therapeutic potential compared to their parent cells, several disadvantages have been reported, including the lack of renewal potential, the loss of some paracrine factors during the use of isolation methods, and the possibility of viral infections transmission and short half-life of exosomes (Takahashi et al., 2013; Babaei and Rezaie, 2021).

### Application Studies of Mesenchymal Stem Cell-Derived Exosomes

Different evidences mentioned in Table 1 have reported the antimicrobial effects of MSCs and MSC-Exos. However, these studies are more limited to animal studies and clinical dates are low. In the evaluation of antimicrobial activity of MSCs in chronic infections associated with biofilm formation, it has been reported that co-administration of MSCs with antibiotics affected both direct and indirect pathways of these cells, such that secretion factors of MSCs inhibited biofilm formation and disrupted the growth of stabilized biofilms (Johnson et al., 2017). It has also been suggested that administration of these cells with antibiotics can have a synergistic effect in reducing a variety of multi-drug resistance (MDR) in bacterial infections (Chow et al., 2020; Russell et al., 2020). Liao et al. reported that hUC-MSC reduced hydrosalpinx, macrophage infiltration, and the expression of IL-10 in the oviduct. Also, they observed that hUC-MSC induced anti-apoptotic effects by reducing the expression level of caspase-3. In addition, it was reported that pregnancy rate increased significantly, and these effects were attributed to the anti-inflammatory and anti-apoptotic properties of hUC-MSC (Liao et al., 2019). In addition, Li et al. (2017) observed that WJ-MSCs restored the epithelial structure of the FT and concentration of TNF was decreased significantly in the treatment group with WJ-MSCs, and they also reported that WJ-MSCs improved the secretion of oviduct glycoprotein and fertility partially in rabbits with chronic salpingitis. Furthermore, Ebrahim et al. (2018) revealed that hUC-MSC-EV alone or in combination with estrogen significantly reduced intrauterine adhesions in female rats due to decrease in inflammatory cytokines (TNF-α, IL-1, IL-6) and fibrotic markers (RUNX2, TGF-β, collagen-I). Also, Ding et al. reported that hUMSC-Exos due to microRNA-17-5P repaired the phenotype and function of the ovary, elevated ovarian cells proliferation, and decreased ROS accumulation in POI mouse model (Ding et al., 2020).

## Conclusion

Experimental studies show that MSCs and MSC-Exos have a high potential for the treatment of inflammatory and microbial diseases. Furthermore, MSC-Exos have similar abilities to their parent cells, which have a high potential for modulating immune responses due to their therapeutic biomolecules. However, the priority of using MSC-Exos compared to cell-based therapy in terms of safety and stability has been reported in several studies. In addition, MSC-Exos induce the phagocytic activity of neutrophils and macrophages and improve the bioenergetics of them to provide the conditions for increasing the survival of these cells and the continuity of their function in bacterial phagocytes. On the other hand, MSC-Exos play an important role in preventing the pathological immune response by interacting with immune cells and reprogramming M1 macrophages to the M2 phenotype and converting Th to Tregs. Therefore, it can be said that MSC-Exos due to these properties can inhibit pathological immune responses during *N. gonorrhoeae* and *C. trachomatis* infections, and in this way MSC-Exos provide the conditions for tissue repair and prevent severe tissue damage during infection.

## Author Contributions

MZ wrote the draft of manuscript. LD, MI, and SM revised the parts of Infectious and infertility. BA read the manuscript and did the final revision and agreed with the final version of the manuscript.

## Conflict of Interest

The authors declare that the research was conducted in the absence of any commercial or financial relationships that could be construed as a potential conflict of interest.

## Publisher’s Note

All claims expressed in this article are solely those of the authors and do not necessarily represent those of their affiliated organizations, or those of the publisher, the editors and the reviewers. Any product that may be evaluated in this article, or claim that may be made by its manufacturer, is not guaranteed or endorsed by the publisher.
